# Reporting bias in trials of volume resuscitation with hydroxyethyl starch

**DOI:** 10.1007/s00508-014-0503-y

**Published:** 2014-03-05

**Authors:** Christian J. Wiedermann

**Affiliations:** Academic Teaching Department of Internal Medicine, Central Hospital of Bozen/Bolzano, Lorenz-Böhler-Street 5, 39100 Bozen/Bolzano (BZ), Italy

**Keywords:** Hydroxyethyl starch, Renal injury, Reporting bias, Hydroxäthylstärke 130/0,40–0,42, Akute Nierenschädigung, Publikationsbias

## Abstract

The possibility of renal damage by hydroxyethyl starch has become the focus of intensive dispute based on the findings of published large trials. The aim of this narrative review is to analyze outcome reporting bias in the literature on volume resuscitation, focusing on selective outcome reporting in published randomized and observational trials with “modern” hydroxyethyl starch as therapeutic intervention. Three recent publications claimed to confirm renal safety of hydroxyethyl starch 130/0.4 for indications in severe sepsis, trauma, and critical illness, respectively. Selective outcome reporting was identified in these studies including underreporting of side effects and change of primary study outcomes. In conclusion, selective outcome reporting bias is identified in recent publications of clinical trials on volume resuscitation with HES.

## Abstract (de)

Die Gefahr der Nierenschädigung durch Hydroxyethylstärke (HES) hat sich zu einem Hauptthema der Diskussion von Ergebnissen rezenter klinischer Studien entwickelt. Ziel dieses Kommentars ist es, Vorhandensein und Art von Publikationsbias in der klinischen Forschung mit Hydroxyethylstärke zu analysieren, wobei der Schwerpunkt auf der selektiven Berichterstattung von Ergebnissen in randomisierten kontrollierten Untersuchungen und Beobachtungsstudien mit „moderner“ Hydroxyethylstärke liegt. Drei aktuelle Publikationen postulierten renale Sicherheit von Hydroxyethylstärke 130/0,4 in den Indikationen schwere Sepsis, Trauma und intensivstationspflichtige Krankheit. Selektive Ergebnisberichterstattung wurde in allen drei Studien identifiziert einschließlich der Unterschlagung von Nebenwirkungen und der Abänderung von vordefinierten primären Studienendpunkten. Publikationsbias in Form selektiver Berichterstattung in mehreren jüngsten Veröffentlichungen klinischer Prüfungen zur Volumentherapie mit HES erschwert die Arbeit von Arzneimittelbehörden, pharmazeutischem und ärztlichem Personal.

## Introduction

Reporting bias is of two types, including, at the study level, absence of a publication because of failure to submit to, or rejection by, scientific journals, and the selective non-reporting of outcomes at the outcome level [1]. On average about half of all clinical studies are fully published [2–6]. This means that only part of the available evidence on a topic is being made public. Furthermore, in various reviews extensive selective reporting in study publications has been found [7–9] including introduction, omission, or change of primary study outcomes [7, 9]. Reporting bias particularly concerns adverse events that are not reported [10–13].

In the fields of perioperative, emergency, and critical care medicine, hydroxyethyl starch (HES) solutions are commonly used for volume resuscitation. In March 2011, 88 publications of Dr. J. Boldt, at one time a prolific German anesthesiologist and pre-eminent authority on the perioperative use of HES, were retracted because he allegedly failed to secure approval from an institutional review board for his studies [14]. In addition, several of his papers were determined to have been fabricated [15]. New meta-analyses assessing outcomes after HES exposure demonstrated no overall effect of HES on survival. However, after exclusion of seven sepsis and trauma studies by Dr. Boldt’s group, significantly increased mortality was shown among HES recipients [16]. Despite existing concerns about the drug’s safety, in particular a nephrotoxic potential that is held responsible for the requirement of renal replacement therapy (RRT) in a significant number of patients receiving HES [17], lack of an association with acute kidney injury continues to be claimed for certain clinical conditions and doses of HES [18]. The arguments in favor of this claim are based on trial data that were published more recently [19–21]. These publications are analyzed here in a narrative review that identifies various types of significant reporting bias.

## Hydroxethyl starch for volume resuscitation

### Clinical use

In the past 15 years, colloids have been increasingly widely used for volume resuscitation in patients with severe acute disease [22]. In Europe, volume resuscitation was achieved preferentially with HES, an artificial colloid derived from plant starch. One of the reasons for the increasing use of HES, despite safety concerns, is that under physiological conditions iso-oncotic HES infusion solutions are more effective volume expanders than isotonic crystalloid solutions such as 0.9 % sodium chloride [23], and furthermore that artificial colloids like HES are associated with a lower cost than the natural colloid albumin [24]. The widespread use of HES has been accompanied by an increase in safety concerns, particularly with respect to kidney damage, bleeding, and severe, persistent, delayed-onset pruritus [25–29]. The risk of these complications became known soon after the introduction of HES as a volume replacement therapy (reviewed in [25]). Marketing authorization for HES was obtained in the late 1960s without the rigorous evaluation of effectiveness and safety in large phase III trials that would be required today. However, as early as 1968 a fall in platelet count and clinical bleeding was reported in a study of HES-treated patients [30]; later, in 1992, a warning about HES and kidney failure was published [31].

Initial strategies to reduce the known risks of HES included redefining the maximum daily dose for different HES preparations [32] and attempting to optimize the crude material used in the manufacture of HES. The resulting changes in pharmacodynamics and pharmacokinetics were expected to improve the tolerability of the substance. Development of lower molecular weight products and alteration of the degree of hydroxyethyl substitution led to “new generation” HES which became part of the marketing strategy by the HES producing companies [33]. “Molar substitution”, the proportion of the glucose rings with at least one hydroxyethyl group, is another feature of HES products; for example, at a substitution of 0.5, 50 % of the glucose rings are substituted with at least one hydroxyethyl group. The original “first generation” HES had a molecular weight (MW) of about 450,000 with a rate of molar substitution of 0.7. The second generation HES had a MW of 200,000 with a substitution of 0.62 or 0.5, and the third generation now has a MW of 130,000 with a substitution of only 0.4. Third generation HES 130/0.4 and 130/0.42 in clinical use is produced from corn starch and potato starch, respectively. For infusion, HES is dissolved in 0.9 % saline or balanced electrolyte solution where part of the chloride is replaced with lactate or acetate.

### Safety concerns regarding “modern” hydroxyethyl starch

First serious doubts about the tolerability of “modern” (second and third) generation HES arose from studies in the intensive care unit (ICU) [34, 35]. In these studies in critically ill patients with severe sepsis, HES with a MW of 200,000 was compared either with gelatin [34] or crystalloid solution [35]. Administration of HES in septic patients was associated with adverse effects on the kidneys that were observed within a follow-up period of 90 days, with the effects being dose-dependent (with high doses of HES there was a trend for a higher rate of death) [35]. The applicability of these results to third generation HES has been strongly challenged. The hypothesis that third generation HES is safer than previous generations of HES, however, was not based on valid data [24, 36]. In 2012, two blinded, randomized, controlled studies, the 6S [37] and the Crystalloid vs. Hydroxyethyl Starch Trial (CHEST) [38] studies were published. Both studies included large numbers of patients, steadily decreasing HES dosages, and follow-up periods sufficiently long (90 days) to observe delayed side effects. In the two studies, potato [37] and corn starch-based [38] third generation HES solutions were evaluated at doses significantly lower than those authorized according to the manufacturers (50 ml/kg body weight/day). After 90 days of follow-up in the 6S study, patients with severe sepsis receiving HES required RRT more often than those receiving Ringer’s acetate solution. There was also an increased incidence of end-stage renal failure and a higher mortality rate, and the median cumulative dose of HES was 44 ml/kg of ideal body weight [37]. In the CHEST study, where most postoperative patients had been admitted without sepsis, there was an increased rate of RRT in the HES than in the normal saline group. In addition, during the first 7 days after admission to the study, the serum creatinine concentrations were significantly higher, and the excreted urine volumes significantly lower, than in the normal saline group. In the CHEST study, which included patients with markedly lower disease severity than the 6S study, mortality was not significantly affected by HES; the median daily dose of HES was 8 ml/kg of body weight [38].

After the publication of these large randomized and blinded studies, meta-analyses confirmed that HES solutions, including third generation HES, exerted negative effects on renal and hepatic function, and coagulation [16, 17, 39–43]. It was concluded that HES should no longer be used in the ICU or in critically ill patients.

On June 14, 2013 in its risk assessment process, the Pharmacovigilance and Risk Assessment Committee (PRAC) of the European Medicines Agency (EMA) concluded that the benefits of HES no longer outweigh the risks and recommended the suspension of all HES marketing authorizations [44]. In the United States of America, the Food and Drug Administration (FDA) issued a “boxed warning” [45].

### Fraudulent Hydroxyethyl starch research affecting the controversy about the clinical use of hydroxyethyl starch

Among opinion leaders and physicians, very different perceptions and treatment algorithms are proposed for volume resuscitation with crystalloids and colloids, inspite of the evolving evidence base described above. The opinions presented are controversial [46]. According to many, successful commercialization by the manufacturers rather than scientific evidence lies behind the widespread use of HES [47]. Furthermore, the controversy is apparently affected by the “Boldt case”, a particularly impressive example of scientific misconduct [14].

## Reporting bias in hydroxyethyl starch clinical research publications

### Case 1

Volume resuscitation with HES is still proposed by some clinicians [48] for various clinical scenarios, including the initial phase of sepsis, despite evidence of safety concerns. This view appears to be based on the CRYSTMAS trial [21], a double blind, prospective, randomized study of 174 septic patients, in whom no significant between-group differences were described following an observation period of 90 days. The trial compared the use of 6 % HES 130/0.4 and 0.9 % saline in severe sepsis to fulfill a postmarketing study commitment issued by the US FDA. Trial outcomes, however, were selectively published [49]. When the more complete data set presented to FDA was evaluated, the results suggested that the use of HES 130/0.4 did not lead to clinically relevant volume savings (the primary outcome parameter), that there were negative effects on kidney function similar to those observed with an older HES solution, and also that there was a 6.8 % increase in 90-day mortality [50]. An obvious limitation of the study was that it was too small to demonstrate a difference in the incidence of renal failure. In the HES group, 24 of 98 patients (24.5 %) developed renal failure compared with 19 of 95 (20 %) in the saline group. After 90 days, 21 patients in the HES group required RRT, almost twice as many as in the saline group (*n* = 11). These study data were not reported in the journal article but have been added to the Voluven Package Insert by FDA [49, 50].

### Case 2

In the randomized, double-blind Fluid-in-Resuscitation-of-Severe-Trauma (FIRST) study, 0.9 % saline was prospectively compared with HES 130/0.4 for initial volume resuscitation in 67 patients with penetrating trauma and 42 patients with blunt trauma [20]. In patients with penetrating trauma, significantly less volume was needed for resuscitation in the colloid group compared with the crystalloid group, and the incidence of renal failure was significantly lower. The FIRST trial included an observation period of 30 days but lacked the power to address renal safety. In the published report, improvement in renal function in patients with penetrating trauma receiving HES 130/0.4 was emphasized. In contrast, an observed increase in transfusion of blood products in HES 130/0.4 recipients with blunt trauma was minimized [51].

In the trial register entry (ISRCTN42061860), prespecified endpoints included two primary and seven secondary endpoints [52]. However, in the published report, three safety endpoints were described that were not included in the original protocol; one of these was acute kidney injury [51]. Thus, there is considerable discrepancy between the prespecified study endpoints and those in the published report.

The CONSORT authors recommend that effect size and confidence interval are given for each primary and secondary outcome [53]. James et al. [20] have not done this but concluded that HES improved renal function. However, serious concerns have been raised on how such a conclusion could have been made, as additional information would have been required, which was not presented in the publication [54].

### Case 3

The retrospective study of Boussekey et al. [19] included 363 patients hospitalized for more than 72 h in the ICU. Of these, 168 patients received HES during their stay and 195 did not [19]. The authors concluded that HES did not induce acute kidney injury because patients in the HES group were more severely ill on admission; however, acute kidney injury incidence and ICU mortality was similar in both groups [19]. This publication has been used to provide evidence that low doses of HES 130/0.4 do not cause kidney failure and cited in the recent meta-analysis on the effects of waxy maize-derived HES 130/0.4 on renal function in surgical patients [55] as well as the Consensus Statement of the European Society of Intensive Care Medicine Task Force on colloid volume therapy in critically ill patients [56]. The publication by Boussekey et al. [19] in *Critical Care* was, however, published with incomplete RRT data. This observation was made because the study results had been previously submitted for publication to another journal. Contents of the preceding manuscript had included the entire set of RRT data which apparently were not presented in the *Critical Care* publication [57, 58].

In the published study by Boussekey et al. [19], RRT data was listed under “Hemofiltration”. However, only 8 patients required RRT according to Table 3 of the paper compared with a total 82 of patients who required RRT in the study’s entire cohort which was initially submitted to another journal. The explanation for this was that Boussekey et al. [19] excluded the majority of their patients before presenting the RRT results. The total population of 363 patients was represented in opening paragraph of their results, in Table 1 and in Figs. 1–3. However, in their results, the incidence of acute kidney injury with regard to HES administration was evaluated by separating the patients into two groups, one with normal kidney function and one with a RIFLE (Risk, Injury, and Failure; and Loss, and End-stage kidney disease) class “risk” on admission [19]. There was no explanation that they separated the patients into two groups only after first excluding the 199 patients, comprising 55 % of the total, who fell into RIFLE categories higher than “risk”. Thus, the RRT data presented in the *Critical Care* publication were based on 164 patients with either normal renal function or minimal renal impairment at baseline, and accounted for only 9.8 % (8/82) of all RRT cases. In contrast, the same publication incorporated outcome data pertaining to all 363 patients for several endpoints, namely, incidence and duration of secondary shock, vasopressor usage, frequency, and duration of mechanical ventilation, and mortality [19]. In the presentation of results to another journal, the same outcome data had been accompanied by the RRT results, in which it was noted that development of acute renal failure was similar in patients with (38/168, 22.6 %) or without (39/195, 20.1 %) HES (*p* = 0.56). However, patients with HES needed RRT more frequently (27.4 vs. 18.5 %, *p* = 0.042), for a longer duration (2.55 ± 5.9 vs. 1.54 ± 5 days, *p* = 0.08), and were more exposed to aminoglycosides (33.9 vs. 24.6 %, *p* = 0.05). Finally, patients with HES had more nosocomial infections (34.5 vs. 22.6 %, *p* = 0.011), a longer ICU stay (20.4 ± 18.9 vs. 15.3 ± 17.5 days, *p* = 0.009), and a nonsignificant ICU mortality increase (31.1 vs. 25.8 %, *p* = 0.26) [57, 58]. Even though it was an observational retrospective study of low-grade scientific evidence, the final publication omitted the RRT data for the full study population and the significant difference in RRT.

With this background, the title of the paper is misleading. The entire data set of the study by Boussekey et al. [19] provides evidence of potential deleterious renal effects associated with starch infusion [57, 58].

## Discussion

Reporting bias is a problem in volume resuscitation research. The increased likelihood for a study of being published when results are positive or favorable results is well known than when negative or unfavorable results were observed [7]. Within a study, selective reporting involves analyses or outcomes [1]. In the three publications discussed here [19–21], reporting bias consisted of selective underreporting of side potential effects [19, 21, 49, 50, 57, 58], selective reporting of analyses [21, 57, 58], and presentation of safety endpoints that were not predefined in the study protocol [20, 51, 53]. In the context of the ongoing debate on the use of HES for volume resuscitation at both clinical and regulatory levels, it is important to make reporting bias public if studies are being used as evidence base regulatory decision making [44, 45, 48, 56].

Industry sponsorship or industry affiliation of authors is associated with positive research outcomes and conclusions [59]. For the CRYSTMAS trial, “Fresenius Kabi was involved in the study design, analysis and preparation of the report” [21]. The FIRST trial “was supported by an unrestricted educational grant from Fresenius-Kabi who also provided the study fluids”; the lead author and two of the five co-authors “have received honoraria and travel support from Fresenius-Kabi” [20]. Boussekey et al. [19] declared that they have no competing interests.

It is concluded that reporting bias is a problem in volume resuscitation research not only because of effects of the retraction of studies by Bold et al. on results of meta-analyses [14, 16] but also because of selective underreporting of adverse events in patients receiving “modern” HES [19–21].
